# A Hybrid Automatic Model for Circle Detection in X-Ray Imagery: A Case Study on Hip Prosthesis Wear

**DOI:** 10.3390/bioengineering13020235

**Published:** 2026-02-17

**Authors:** Mehmet Öztürk, Yahia Adwan

**Affiliations:** Department of Electrical and Electronics Engineering, Karadeniz Technical University, Trabzon 61080, Turkey; mehmetozturk@ktu.edu.tr

**Keywords:** circle detection, hybrid deep learning, computer vision, YOLOv5, geometric feature extraction

## Abstract

This study presents a fully automatic hybrid framework for circle detection and geometric feature extraction from anteroposterior (AP) X-ray images. Detecting circular structures in X-ray imagery is challenging due to low contrast, noise, and metal-induced artifacts, which often limit the robustness of purely learning-based or purely geometric approaches. To address these challenges, a hybrid deep learning and computer vision pipeline is proposed that combines data-driven region localization with robust geometric fitting. A YOLOv5-based detector is first employed to identify a compact region of interest (ROI) containing circular components. Within this ROI, edge-based processing using Canny detection is applied, followed by an Edge-Snap refinement stage and robust RANSAC-based circle fitting with a Hough-transform fallback to ensure anatomically plausible circle estimation. The resulting circle centers and radii provide stable geometric parameters that can be consistently extracted across images with varying contrast, noise levels, and prosthesis appearances. The applicability of the proposed framework is demonstrated through a case study on hip prosthesis wear analysis, where the automatically detected circle parameters are used to compute medial, superior, and resultant displacement components using established two-dimensional radiographic formulations. Experimental evaluation on AP hip radiographs shows that the YOLOv5 detector achieves high ROI localization performance (mAP@0.5 = 0.971) and that the hybrid pipeline produces consistent circle parameters across longitudinal image sequences. Overall, the proposed method provides an end-to-end automatic solution for robust circle detection in X-ray imagery, with hip prosthesis wear presented solely as a case study without clinical or diagnostic claims.

## 1. Introduction

### 1.1. Application Background and Problem

Total hip arthroplasty (THA) is widely regarded as one of the most successful operations in orthopedic surgery, providing substantial pain relief and functional restoration for patients with end-stage hip disease. Large registry-based analyses and meta-analyses have shown that modern hip replacements can remain in situ for 20 to 25 years in a substantial proportion of patients, yet a considerable number of implants will eventually fail during the lifetime of younger and more active patients who increasingly undergo THA [1,2]. A contemporary THA construct typically consists of a metallic femoral stem, a modular femoral head, and an acetabular component that includes a metallic shell and a bearing liner.

**Figure 1:**
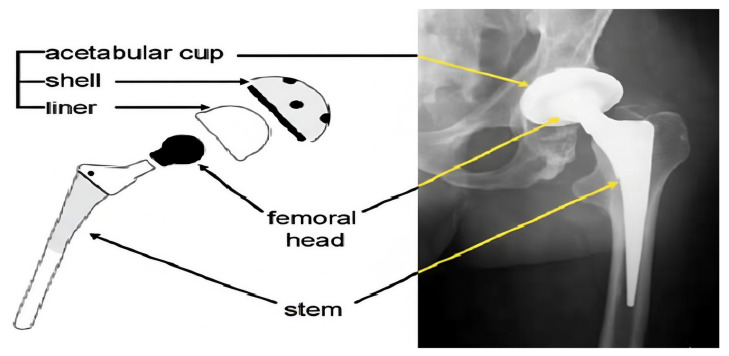
Hip Prosthesis Components.

Bearing couples most often combine a cobalt-chromium or ceramic femoral head with a polyethylene liner, frequently using highly cross-linked polyethylene (HXLPE) or antioxidant-stabilized formulations in order to improve wear resistance [3,4]. The main components of a THA construct are illustrated in Figure 1. Advances in materials science and implant design have significantly reduced volumetric wear compared with earlier generations of polyethylene, but wear has not been eliminated. Recent clinical studies in young and active cohorts still report measurable linear wear of HXLPE and demonstrate that even low annual penetration rates may accumulate over decades to produce clinically relevant changes in component position [4,5,6].

Prosthetic wear particles and interfacial micromotion drive a biological cascade that can result in periprosthetic osteolysis, progressive loss of bone stock, and aseptic loosening of one or both components [2,3]. Despite improvements in fixation and bearing technology, polyethylene wear and osteolysis remain key mechanisms underlying late failure and revision surgery, particularly in patients with long life expectancy and high activity levels [2,4,7]. Case series and reports continue to describe catastrophic osteolysis, eccentric migration of the femoral head within the acetabular cup (AC), and massive cavitary defects around otherwise well-fixed components when wear is not detected and addressed in a timely fashion [7,8]. These complications increase the technical difficulty of revision surgery and are associated with higher costs, longer operative times, and worse functional outcomes compared with uncomplicated primary THA [2,4]. Consequently, early recognition of excessive wear and subtle radiographic signs of osteolysis is critical for planning timely intervention before extensive bone loss occurs.

International guidelines and systematic reviews emphasize the importance of structured long-term follow-up after THA, yet they also highlight the lack of robust evidence and the substantial variation in current practice [9,10]. Some expert-based recommendations advocate periodic clinical and radiographic surveillance beyond the early postoperative period, but there is no universal consensus on the optimal frequency or duration of follow-up for different implant types and risk profiles [9,10]. Health service analyses from the United Kingdom and other countries show that routine arthroplasty follow-up is costly and inconsistently implemented, with many centers reducing or discontinuing mid- to late-term visits in order to cope with resource constraints [10]. At the same time, systematic reviews of follow-up pathways stress that plain anteroposterior (AP) pelvis or hip radiographs remain the primary imaging modality for detecting asymptomatic component migration, radiolucent lines, and cavitary osteolysis in most health systems [9,11]. In this context, reliable quantitative assessment of wear using standard radiographs is a key unmet need for both clinicians and policy makers who must balance patient safety with the pressure to rationalize follow-up.

Radiographic wear measurement can be performed with highly accurate three-dimensional (3D) methods such as radiostereometric analysis (RSA), but RSA requires specialized equipment, marker insertion, and dedicated imaging protocols that are rarely available in routine clinical practice [2,11]. Consequently, the vast majority of centers rely on two-dimensional (2D) methods that infer polyethylene wear from serial conventional radiographs. These approaches typically estimate the centers and radii of the femoral head and the AC, and derive linear or vectorial wear components from changes in head position relative to the AC over time [11,12]. Experimental work has demonstrated that 2D wear estimation is sensitive to pelvic orientation, radiographic projection, and image quality, while clinical studies confirm that manual or semi-automatic measurements exhibit non-negligible interobserver and intraobserver variability [11,12]. Accurately identifying the bony landmarks and the circular outlines of the femoral head and AC on noisy, variably exposed AP radiographs is laborious even for experienced readers and becomes increasingly challenging when implants are superimposed on complex anatomy, when metal artifact is present, or when multiple follow-up series must be compared [11,12]. As a result, radiographic wear analysis is often restricted to research settings or small cohorts and is rarely integrated into routine follow-up at scale, despite its potential to detect early migration and osteolysis.

### 1.2. Conventional Radiographic Wear Measurement and Limitations

Conventional radiographic wear analysis in THA is fundamentally based on measuring femoral head penetration relative to the AC on serial AP radiographs. In classical 2D techniques such as those described by Charnley, Livermore, and Dorr and Wan, the outlines of the femoral head and AC are approximated as circles on each radiograph, the centers of these circles are determined manually, and linear wear is inferred from the change in head center position relative to the AC center over time. Although the original descriptions of these methods are several decades old, recent clinical studies and methodological reviews still use these frameworks, or their derivatives, as reference standards when reporting in vivo polyethylene wear and femoral head migration in contemporary cohorts with HXLPE and dual mobility bearings [13,14,15,16,17].

Subsequent computer-assisted methods, including the Martell technique and the 3D Devane or PolyWare-based analysis, extend this geometric principle by digitizing implant edges and reconstructing head penetration vectors in 2D or 3D. These systems typically fit parametric models to the projected head and cup contours, correct for radiographic magnification, and compute superior and center-to-center (medial)wears, sometimes with an additional anterior component when biplanar or model-based reconstructions are available. In recent work, such techniques have been used to quantify very low HXLPE wear rates in long-term RSA studies, to validate the stability of acetabular constructs, and to compare different bearing materials or head sizes using linear penetration and head migration as primary endpoints [13,14,15,16,17]. More recently, novel vector-based wear algorithms have been proposed that modify or replace the traditional Martell and Devane calculations, but these approaches still rely on the requirement that head and cup geometry be reliably identified on standard radiographs [13,18].

Despite their widespread use, these conventional methods share important limitations that restrict their applicability in routine clinical follow-up and large-scale research. First, most Charnley or Livermore type techniques are purely 2D and assume that wear occurs in the plane of the AP radiograph, which means that out-of-plane components or complex 3D head trajectories are not fully captured. Even in more advanced Devane or PolyWare-based 3D reconstructions, accuracy depends strongly on standardized imaging protocols, consistent pelvic positioning, and high-quality radiographs, which are difficult to guarantee in everyday practice [18,19]. Second, all of these methods require meticulous manual or semi-automatic identification of the femoral head and AC outlines on each radiograph. Manual landmark placement is time-consuming, operator-dependent, and subject to interobserver and intraobserver variability, particularly in the presence of metal artifact, suboptimal contrast, or complex anatomy [13,15,17]. Third, because each radiograph must be exported to dedicated software and processed through a relatively labor-intensive workflow, radiographic wear analysis is usually reserved for specialized centers, research projects, or small to medium-sized cohorts rather than being applied systematically across entire arthroplasty registries or routine outpatient follow-up programs [16,19]. These constraints mean that, although classical methods by Charnley, Livermore, Dorr and Wan, Martell, and Devane have provided the conceptual foundation for quantitative wear assessment, there is still a clear need for more automated, robust, and scalable pipelines that can extract the same geometric information directly from routine clinical radiographs with minimal user intervention.

### 1.3. Deep-Learning-Based Hip Prosthesis Analysis: Related Work

The expanding clinical burden of late complications after THA, including polyethylene wear, osteolysis, and aseptic loosening, together with the high expectations for long-term survivorship in younger and more active patients, has renewed interest in more objective and scalable image-based surveillance strategies [1,2,3,4,5,6,7,8]. At the same time, clinical guidelines and health service analyses emphasize that routine follow-up after THA remains heterogeneous, resource-constrained, and largely dependent on qualitative interpretation of AP radiographs despite the recognized value of quantitative radiographic wear and migration measurements [9,10]. Conventional 2D and 3D radiographic methods, including RSA and its 2D derivatives, can provide highly accurate estimates of head penetration and cup migration, but they require specialized protocols and remain sensitive to pelvic orientation, projection geometry, and manual landmark identification [11,12,13,14,15,16,17,18]. In this context, deep learning and other artificial intelligence (AI) techniques have recently been explored to automate the analysis of plain hip and pelvis radiographs after THA, with the aim of reducing observer dependence and enabling more consistent large-scale assessment.

Most existing AI applications in THA radiography focus on detection, classification, or risk stratification rather than explicit geometric reconstruction of femoral head and AC parameters. Several groups have developed convolutional neural network (CNN) models that classify implant type or manufacturer on AP radiographs, showing that deep learning can recognize specific THA designs with very high accuracy and outperform experienced surgeons in implant identification tasks [20,21,22]. These systems are particularly attractive for preoperative planning in revision surgery, where knowledge of the exact implant model is critical yet often difficult to obtain from the clinical record. Other work has applied CNN-based pipelines to detect or predict the mechanical loosening of hip implants on plain radiographs. Borjali et al. trained a deep CNN on AP hip radiographs and reported that their model could diagnose loosening with higher sensitivity than an expert orthopedic surgeon at comparable specificity [23], while a recent systematic review and meta-analysis concluded that machine-learning approaches on plain radiography show promising accuracy for detecting loosening around hip and knee arthroplasty. However, further validation is required before widespread clinical adoption [24]. More recently, Masciulli et al. proposed radiograph-based AI models that incorporate multiple follow-up images to predict hip implant failure, highlighting the potential of temporal deep-learning representations for longitudinal risk prediction on X-ray series [25].

A second line of work has focused on automated measurement of radiographic outcome parameters known to influence instability and wear. Rouzrokh et al. introduced a deep-learning tool that automatically measures AC inclination and version angles on postoperative AP radiographs, demonstrating that a CNN-based system can match or exceed manual measurements of cup orientation and substantially reduce reading time [26]. In a complementary study from the same group, a CNN was trained on a large cohort of postoperative AP pelvis radiographs to classify patients according to their risk of dislocation following primary THA, again showing that deep learning can exploit subtle radiographic features to provide clinically meaningful risk stratification that may inform follow-up pathways and rehabilitation protocols [27]. Beyond these single-center developments, recent multi-center work has demonstrated that implant identification and hardware classification on pelvis and hip radiographs can generalize across institutions when models are trained on sufficiently large and diverse datasets, which further supports the feasibility of integrating AI tools into routine THA image analysis workflows [21,22].

More recent studies have begun to bridge implant identification with X-ray-based outcome assessment. Musbahi et al. described an AI framework that combines hip prosthesis identification with quantification of radiographic outcome measures on postoperative radiographs, illustrating how CNN-based systems can simultaneously recognize implant configuration and extract clinically relevant measurements from THA images [20]. Kim et al. synthesized the emerging evidence on AI-based detection of prosthetic loosening and concluded that machine-learning models trained on plain radiographs achieve good sensitivity and specificity across both hip and knee arthroplasty, while emphasizing that most current algorithms are still designed for binary classification and have not been extended to full quantitative wear or migration measurements [24]. Taken together, these studies demonstrate that deep learning on AP hip and pelvis radiographs can successfully automate implant detection, design classification, cup orientation measurement, and prediction of dislocation or loosening risk after THA. However, they typically operate at the level of global labels or angular measurements and do not explicitly recover the centers and radii of the femoral head and AC circle that underlie conventional 2D wear formulas. To date, no deep-learning framework provides end-to-end extraction of these geometric parameters from routine AP radiographs in a form that can directly substitute for established radiographic wear measurement methods, which motivates the hybrid detection and circle-fitting approach proposed in this work.

### 1.4. Motivation and Contributions of This Work

Despite substantial improvements in implant design, bearing materials, and fixation techniques, long-term follow-up studies continue to show that wear-related osteolysis and aseptic loosening remain leading causes of late failure and revision after THA, especially in younger and more active patients. Recent work on HXLPE liners and alternative bearing compositions confirms that linear penetration and head migration are reduced but not eliminated, and that small annual wear rates can still accumulate over one or two decades to produce clinically relevant changes in component position [13,14,15,16,17,18,19,28]. At the same time, health service and policy analyses highlight that routine THA surveillance is resource-intensive, that there is considerable variation in how often patients are recalled, and that the clinical value of systematic follow-up radiographs is still debated in the context of increasing workload and financial pressure [29]. High-precision 3D techniques such as RSA provide gold-standard wear and migration data, but they require specialized markers, imaging protocols, and software, which limits their use to dedicated research centers [14,16,28]. As a result, the vast majority of institutions continue to rely on conventional 2D AP radiographs and manual or semi-automatic measurements of femoral head and AC position over time, despite well-documented interobserver variability and the considerable time required to perform these analyses on large cohorts [18,19,28].

Parallel to these developments, there has been a rapid expansion of AI and deep learning applications in musculoskeletal imaging and orthopedic surgery. Recent narrative reviews and primers emphasize that computer vision models are increasingly used to detect fractures, classify implants, and quantify alignment or morphology on radiographs, with the dual aim of improving diagnostic accuracy and reducing radiologist workload [30,31,32,33]. In the specific context of THA, CNN-based models have been applied to identify hip and knee prosthesis designs, flag mechanical loosening, populate radiographic registries, recover cup inclination and version, and predict dislocation or failure risk from postoperative AP images [34,35]. Deep-learning systems have also been proposed for fully automated measurement of lower extremity alignment and other geometric parameters on long-leg radiographs, demonstrating that landmark-based quantitative analysis can be reliably automated in data-constrained clinical settings [32,33,34,35]. However, existing hip-specific AI tools usually operate at the level of global labels, implant classes, or angular measurements and do not output the precise circle centers and radii of the femoral head and AC that underpin established 2D radiographic wear formulas. They therefore do not directly replace classical wear methods that require explicit reconstruction of the head within the AC on serial AP radiographs.

This work is motivated by the gap between the clinical need for robust, scalable quantification of hip prosthesis wear on routine radiographs and the current focus of AI methods on detection, classification, or high-level risk prediction. Building on the clinical and methodological literature, our goal is to design a framework that operates directly on standard AP hip radiographs, recovers the geometric primitives required for medial, superior, and resultant wear components, and is suitable for integration into large-scale follow-up workflows. Concretely, the main contributions of this study are as follows. First, we propose a fully automatic hybrid pipeline that combines YOLOv5-based localization of a compact region of interest (ROI) around the femoral head and AC with an edge-based refinement stage and robust RANSAC-driven circle fitting, including a Hough transform fallback, to estimate anatomically plausible circles for both components under challenging imaging conditions. Second, we show how the resulting circle centers and radii can be transformed into standardized medial, superior, and resultant 2D wear vectors using established radiographic formulas, thereby bridging the gap between deep-learning outputs and conventional wear metrics that are familiar to clinicians and researchers. Third, we evaluate the framework on AP radiographs, including serial follow-up images, and demonstrate that the automatically derived wear trajectories qualitatively track expected patterns of femoral head migration within the AC. Taken together, these contributions position the proposed method as a step toward practical, geometry-aware AI tools that can support routine THA surveillance and large-scale outcome studies by automating the most labor-intensive components of radiographic wear analysis.

## 2. Materials and Methods

In this study, all hip prosthesis X-rays were first normalized to a common intensity and size range, allowing images with different original resolutions to be processed within a unified training pipeline. Using a custom Python-based annotation tool, the Circle Selector, an experienced reader manually delineated two circles on each image: one for the femoral head and one for the AC. After contrast enhancement with bilateral filtering, histogram equalization, and Canny edge detection, the relevant contours were made more visible. These circular annotations were converted to a YOLO-compatible format and expanded via data augmentation, including random rotations, flips, scaling, and intensity variations, increasing the effective training set from 100 original radiographs to more than 10,000 augmented samples. A YOLOv5s model was then trained to localize a compact ROI around the prosthesis and to provide an initial box-based approximation of the femoral head and AC, which was subsequently converted into circular proposals. Within this region, an edge-driven refinement stage combined an Edge-Snap optimization, robust RANSAC-based circle fitting, and a Hough-transform fallback to ensure that the final circles were correctly aligned to the anatomical boundaries of the femoral head and AC, even in noisy or low-contrast images. Finally, the resulting normalized centers and radii were used together with established planar formulas for medial and superior wear to compute the overall resultant wear magnitude, focusing on these directions because they correspond to the dominant loading axes and therefore to the most clinically relevant patterns of polyethylene wear.

### 2.1. Clinical and Imaging Dataset Description

The proposed framework was developed and evaluated on a curated set of 100 anonymized AP hip X-ray images containing THA. The radiographs were obtained from open-access radiographic references. In each image, both the femoral head and the AC were clearly visible, allowing two target structures to be annotated per radiograph, yielding 200 circular annotations in total. All radiographs were converted to grayscale, intensity-normalized, and rescaled to a common spatial dimension; hence, images with different original resolutions could be processed within a single pipeline. To reduce overfitting, extensive data augmentation was then applied to the annotated images and their corresponding labels, including random in-plane rotations, horizontal flips, mild scaling and translations, and controlled variations in intensity and contrast. These operations substantially increased the number of available training samples, yielding an augmented pool of more than 10,000 images. For model development, this augmented dataset was subsequently randomly partitioned at the image level into training (70%), validation (15%), and test (15%) subsets, ensuring that each split contained a diverse range of prosthesis types, orientations, and radiographic appearances. In addition to the internally partitioned dataset used for model training and validation, a separate external test subset was employed solely for qualitative and quantitative evaluation. 

### 2.2. Ground-Truth Annotation, Pre-Processing, and Normalization

**Figure 2:**
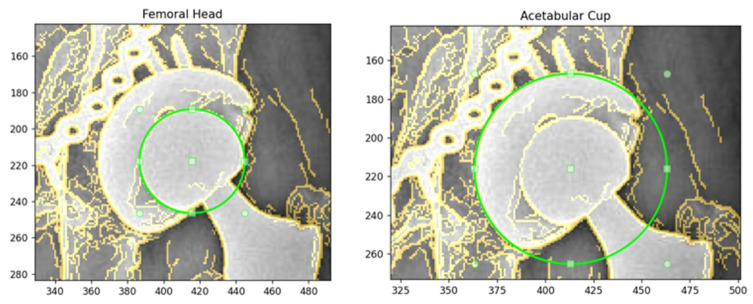
Manual Circle Selector annotations for femoral head and acetabular cup (AC).

All radiographs were annotated using a Python (v3.9) tool termed Circle Selector, which provides an interactive graphical interface for manual delineation of circular structures on AP hip X-ray images. For each radiograph, the femoral head was first selected, followed by the AC, by adjusting a draggable circle to ensure that it aligned with the visible cortical contour. In cases where metal artifacts, screw heads, or partial loss of the outline were present, the circle was centered on the most continuous and anatomically plausible boundary, and any missing segments were interpolated geometrically to preserve a smooth spherical or hemispherical shape. The AC radius was always constrained to exceed the femoral head radius in order to reflect true prosthetic geometry. An illustration of the manual annotation process generated using the Circle Selector tool is shown in Figure 2.

(1) $$r_{\left\{AC\right\}}>r_{\left\{femoral\right\}}$$

Before annotation, each image was subjected to basic contrast and edge enhancement to facilitate contour visibility. Local contrast was improved using contrast-limited adaptive histogram equalization (CLAHE), noise was reduced with bilateral filtering that preserves edges, and a Canny edge detector was applied to produce a stable edge map. The Canny thresholds were determined adaptively from the image intensity distribution: letting v denote the median gray level and σ=0.33, the lower and upper thresholds $T_{\mathrm{low}}$ and $T_{\mathrm{high}}$ were computed as follows:

(2) $$T_{low}=\;\left(1\;-\;\sigma \right)\times v$$

(3) $$T_{high}=\left(1+\sigma \right)\times v$$

This adaptive strategy enabled the edge detector to remain robust across radiographs with varying exposure levels and contrast. The resulting edge map was overlaid semi-transparently on the radiograph to guide circle placement. Each annotated radiograph was saved as a text file with one line per structure using a YOLO-style circular representation of the following form:

(4) cls x y r

where *cls* ∈ {0, 1} denotes the class label (0 for femoral head, 1 for AC), and *x*, *y*, and *r* denote the circle center and radius. To make annotations resolution-independent, the center coordinates and radius were converted from pixel units $\left(x_{px},y_{px},r_{px}\right)$ to normalized values in the range [0,1]. For an image of width W and height H, the normalized parameters were computed as follows:

(5) $$x\;=\frac{X_{px}}{W},\;y\;=\frac{Y_{px}}{H},\;r\;=\frac{r_{px}}{\frac{W+H}{2}}$$

This normalization ensured consistent geometric representation across images with different sizes and aspect ratios and provided a unified interface between the manual annotations, the YOLOv5-based localization network, and subsequent circle-fitting and wear computation stages.

For compatibility with conventional box-based YOLO training, each circular annotation was also converted into an equivalent bounding box without altering the circle center. If (x,y,r) are the normalized circle parameters and $\left(x_{box},y_{box},w,h\right)$ the resulting bounding-box center, width, and height, the conversion was carried out as follows:

(6) $$x_{box\left(px\right)}=\;x_{circle},\;y_{box\left(px\right)}=\;y_{circle}\;$$

(7) $$\;w=\frac{2\;r_{px}}{W},\;h=\frac{2\;r_{px}}{H}$$

A single-step normalized expression can be established as follows:

(8) $$w_{box\left(norm\right)}\;=\;r_{norm}\left(1\;+\frac{H}{W}\right)$$

(9) $$h_{box\left(norm\right)}=r_{norm}\left(1+\frac{W}{H}\right)$$

This ensured that each bounding box precisely circumscribed the corresponding circle in the original pixel space. Together, these normalized circular and rectangular representations formed the ground-truth targets for training and evaluating the hybrid framework in subsequent stages.

### 2.3. Data Augmentation Strategy

On-the-fly data augmentation was used during YOLOv5 training to increase appearance variability while preserving anatomically plausible prosthesis geometry. Augmentations were applied exclusively to the training split; validation and test images were kept unchanged to ensure that the reported metrics reflected performance on non-augmented data. From a geometric perspective, we adopted a deliberately conservative policy tailored to AP hip radiographs. Large rotations, perspective warping, vertical flips, and shearing were disabled to avoid unrealistic projection geometries or inversion of the superior–inferior axis. Instead, each training image could undergo small in-plane rotations of up to approximately ±2°, isotropic scaling within a narrow range (about ±10% of the original size), and translations of up to 5% of the image dimensions, all sampled at random per iteration. A low-probability horizontal flip (*p* ≈ 0.1) was enabled to exploit the left–right symmetry of hip prostheses while maintaining the clinical interpretability of femoral head and AC relationships. These mild geometric transformations were sufficient to expose the detector to variation in prosthesis orientation and positioning without distorting the circular morphology of the femoral head and AC. Photometric augmentation was likewise kept minimal and physically motivated. Since all images were grayscale X-rays, color jittering was disabled, and only controlled changes in global intensity were allowed. In practice, this was implemented through YOLOv5’s brightness (value) jitter with a small amplitude (up to ±10%) together with the contrast and edge-enhancement pre-processing described in the previous subsection, which together emulate differences in exposure and post-processing across acquisitions. No synthetic noise injection or aggressive contrast stretching was used, as these tended to produce visually implausible radiographs in preliminary experiments. Finally, we retained YOLOv5’s mosaic augmentation at a low probability (mosaic ≈ 0.1) to increase the variety of relative scales and surrounding anatomical context seen during training, while disabling mixup and copy–paste augmentations to avoid unphysical blending of overlapping prostheses. Overall, this augmentation strategy approximately expanded the effective training set from 100 images to more than 10,000 distinct training samples over the course of optimization, improving generalization to AP hip radiographs while maintaining the anatomical integrity required for accurate circle-based wear estimation.

### 2.4. YOLOv5 Implementation, Localization Pipeline, and Evaluation Metrics

#### 2.4.1. Rationale for the Hybrid YOLO–Geometric Pipeline

The combination of YOLOv5 with Edge-Snap refinement and RANSAC/Hough-based circle reconstruction was chosen deliberately rather than as a purely technical implementation choice. YOLOv5 is employed only to provide a coarse and robust ROI localization and an initial geometric hypothesis, which substantially reduces the search space and improves computational efficiency without requiring a large, annotated dataset. However, instead of relying on the CNN output as the final estimator, the anatomical parameters are determined through deterministic geometric processing. Edge-Snap alignment constrains the solution to the true acetabular and femoral rim boundaries, while RANSAC circle fitting provides robustness against outliers, metallic artifacts, and incomplete contours. The Hough-transform fallback ensures stability in cases where edge continuity is limited. This hybrid design combines the contextual awareness of deep learning with the accuracy and interpretability of analytic geometry, resulting in a method that is both data-efficient and anatomically reliable.

#### 2.4.2. Model Training Pipeline

All deep-learning components were implemented in Python using the open-source YOLOv5 framework and PyTorch (v2.5.1). All experiments were executed on a single GPU, and random seeds were fixed to 42 to enhance reproducibility. Training was conducted on an NVIDIA RTX-3070 GPU using PyTorch (v2.5.1) CUDA (v12.1) build and CUDA Toolkit (v12.6). The conventional image-processing stages of the pipeline, including edge detection, Hough transforms, and RANSAC-based circle fitting, were implemented using standard image-analysis methods. The detection head was configured for two classes (femoral head and AC), and grayscale radiographs were replicated across three channels to satisfy the network input format. All images were resized to an effective input resolution of 640 × 640 pixels using rectangular training. The dataset was partitioned into training, validation, and test subsets containing approximately 70%, 15%, and 15% of unique radiographs, respectively. The effective training variability was further increased through on-the-fly augmentation.

Training was performed using the adaptive moment estimation with weight decay (AdamW) optimizer with an initial learning rate (LR) of 0.01. A short warm-up phase of three epochs was applied to stabilize early optimization. Loss weights for bounding-box regression, objectness, and classification followed the YOLOv5 defaults with mild dataset-specific rebalancing, and Focal Loss (γ = 0) was disabled. To reduce over-confident predictions and improve generalization, label smoothing with a factor of 0.05 was enabled. The batch size was selected automatically using the YOLOv5 AutoBatch routine, which identifies the largest batch that fits in GPU memory. Training proceeded for up to 400 epochs with early stopping (patience = 80) based on validation mAP, and intermediate model weights were saved every 25 epochs. A summarized overview of the training strategies, together with their objectives and underlying rationale, is provided in Table 1.

To reduce over-fitting to radiographs from a single institution, a two-stage fine-tuning schedule was adopted. In the first stage, the backbone layers up to the 10th module were frozen, and only the higher-level detection layers were optimized for 100 epochs, allowing the network to adapt its final representation to the hip-prosthesis domain while preserving generic low-level features. In the second stage, all layers were unfrozen, and training was resumed from the best checkpoint of Stage 1 for the remaining epochs using a reduced effective LR, enabling global refinement of feature representations while maintaining training stability. All training was performed with cached images in RAM to minimize I/O overhead.

#### 2.4.3. Object-Detection Metrics

Performance of the detector was quantified using standard metrics derived from true positives (TP), false positives (FP), and false negatives (FN). For a given confidence threshold t, precision and recall are defined as follows:

(10) $$Precision\left(t\right)=\frac{TP\left(t\right)}{TP\left(t\right)+FP\left(t\right)}$$

(11) $$Recall\left(t\right)=\frac{TP\left(t\right)}{TP\left(t\right)+FN\left(t\right)}$$

The F1-score, representing the harmonic mean of precision and recall, is computed as follows:

(12) $$F1\left(t\right)=2\;\times \;\frac{Precision\left(t\right)\times Recall\left(t\right)}{Precision\left(t\right)+\;Recall\left(t\right)}$$

Bounding-box overlap is quantified using the Intersection-over-Union (IoU), defined for a predicted region $B_{p}$ and ground-truth region $B_{g}$ as follows:

(13) $$IoU\left(B_{p},\;B_{g}\right)=\frac{\left(\left|B_{p}\cap \;B_{g}\right|\right)}{\left(\left|B_{p}\cup \;B_{g}\right|\right)}\;$$

A detection is counted as a true positive when its IoU exceeds an evaluation threshold τ. Average Precision at IoU threshold τ is computed as the area under the Precision–Recall curve:

(14) $$\;{Average\;Precision}_{\tau }=\;\int_{0}^{1}Precision\left(r\right)\;dr$$

where r denotes recall and k indexes the two object classes (femoral head and AC). mAP over the two classes is then established as follows:

(15) $$mAP_{\tau }=\;\left(\frac{1}{2}\right)\Sigma_{\left\{k=1\right\}}^{2}Average\;Precision$$

In addition to PR curves, model calibration was evaluated using confidence–recall and confidence–precision plots, which illustrate how prediction confidence relates to localization performance. These curves are generated by sweeping the confidence threshold from 0 to 1 and recomputing TP, FP, and FN counts. Together, these metrics provide a comprehensive characterization of detector behavior, including IoU, ranking quality (Average Precision/mAP), and confidence stability (precision–recall–confidence curves).

#### 2.4.4. ROI Localization Using YOLOv5

**Figure 3:**
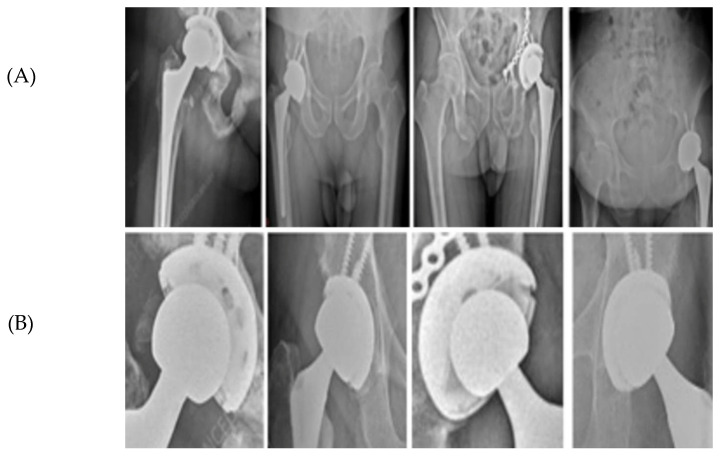
ROI extraction from hip prosthesis X-rays. (**A**) AP pelvic radiographs containing a hip prosthesis. (**B**) Automatically extracted Regions of Interest (ROIs) generated by the YOLOv5 localization stage. Each ROI corresponds to the model’s predicted implant region and is forwarded to the subsequent edge-based refinement and circle-fitting pipeline (RANSAC and Hough-based detection). This step ensures that only implant-focused, anatomically relevant regions are processed during circle estimation.

YOLOv5 was employed as an initial localization module to extract a coarse ROI encompassing the hip prosthesis, as illustrated in Figure 3. Unlike standard multi-class detection pipelines in which bounding boxes directly represent femoral head or AC locations, the detector in this study was configured to identify the prosthesis region as a whole. The output of this stage is therefore a single, high-confidence ROI bounding the implant complex rather than individual anatomical components.

This ROI is subsequently cropped at full resolution and passed to the circle-refinement pipeline (Section E), where component-level delineation is performed. Within the ROI, candidate femoral head and AC circles are generated using RANSAC-based fitting, with a Hough-transform fallback for cases of insufficient edge continuity. All candidates are then subjected to Canny-based edge validation and anatomically constrained filtering to ensure geometrically and biomechanically plausible selection of the final two circles. In this framework, the ROI serves purely as a spatial prior that restricts the search space, while the accurate identification of the femoral head and AC is achieved exclusively through the model’s geometric refinement stages.

### 2.5. Circle Refinement and Hybrid Framework

After YOLOv5 has localized a prosthesis ROI containing the femoral head and the AC, this subsequent stage converts the coarse detection into two accurate circles that follow the true implant outlines. In this hybrid stage, the deep-learning output is only used to define a plausible ROI and an initial circular guess. Meanwhile, the final femoral head and AC parameters are obtained through deterministic edge-based refinement, robust circle fitting, and anatomically constrained selection.

The first step is mapping each radiograph’s YOLOv5 bounding box for the femoral head or AC back to the original image resolution and converting it into an initial circular proposal using the box center as a circle center and the average of the box width and height as the initial radius. Around each detection, at original resolution, an ROI corresponding to the predicted implant region is defined with a small safety margin, which ensures that all relevant edge information around the implant is present. All refinement steps operate within this ROI region, reducing the computational load while preserving full spatial detail around the prosthesis.

We adopt a two-step edge-driven strategy for femoral-head refinement. First, an edge-snap procedure samples radial intensity and edge profiles around the circle center over many angles and radii within a narrow annulus. A combined edge signal is created from the CLAHE-enhanced and sharpened ROI using both Canny edges and gradient magnitudes. The algorithm identifies, for each radial direction, the radius at which the signal is maximal, and these radii are pooled across angles using a robust weighted median to obtain an updated radius that closely adheres to the cortical boundary. Second, high-response edge points in a thin ring around this snapped radius are passed to a RANSAC-based circle fitter. Multiple circles are hypothesized from random triplets of edge points, and inliers are defined by their distance to the circle. The best hypothesis is subsequently refined with a least-squares fit, yielding a well-aligned femoral-head circle, and is robust against spurious responses from noise, screw heads, or partial occlusion.

The AC is refined using a more elaborate search because its rim is often only partially visible and must remain geometrically consistent with the femoral head. Starting from the ROI-based AC detection, a pool of candidate circles is generated by slightly shifting the circle center within the predicted region and scaling the radius within a physiologically plausible range. For each candidate, the radius is first re-aligned to local edges using a 360° edge-snap procedure similar to that used for the femoral head, and can then be further refined with RANSAC. The quality of each resulting circle is quantified by a composite score that combines several image-driven criteria in the ROI predicted by YOLOv5: the fraction of high-response edge points that lie within a narrow tolerance of the fitted radius, the overall angular coverage of these supporting points together with the length of the longest continuous supporting arc, the agreement between local gradient directions and the outward normal of the circle, proximity of the circle center to the YOLOv5 ROI center, and the proportion of supporting edge points that lie outside the femoral-head circle rather than cutting through it. Circles that are strongly supported by continuous, well-aligned edges that surround the head within the predicted implant region naturally obtain higher scores.

On top of these image-based scores, a final anatomically constrained selection is applied such that the surviving femoral-head and AC circles correspond to a realistic total hip prosthesis configuration. The radius of the AC must be larger than that of the femoral head, reflecting the true geometry of a head seated within a larger cup. The two circles cannot intersect or touch; a small but non-zero joint-space gap must remain between them to simulate a physiologically plausible polyethylene layer. The center of the AC must envelop the femoral head from the outside; candidates whose center drifts towards the contralateral hip or clearly outside the pelvic region are rejected. Around each selected circle, the local Canny-based edge density must be larger than a minimum threshold to ensure that purely noise-driven or weakly supported solutions are discarded.

**Figure 4:**
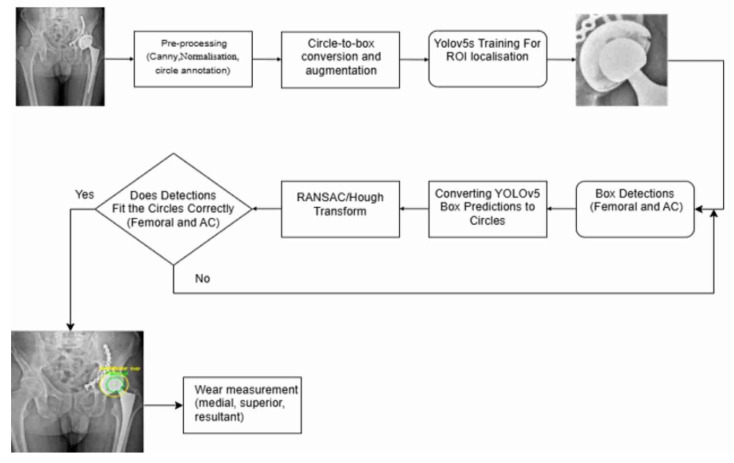
End-to-End workflow: From ROI localization to circle refinement and wear computation.

A summary of the full hybrid pipeline, including ROI localization, edge-based refinement, circle fitting, and wear computation, is illustrated in Figure 4. After the candidate femoral-head and AC circles are generated through edge-snap refinement and RANSAC fitting, they are subjected to several image-based and anatomical plausibility checks. Candidates that closely follow screw paths, metal plates, or glare artifacts typically exhibit unstable radii and poor directional continuity; such circles are therefore rejected by the combined edge-support and stability criteria. This anatomically informed filtering step systematically removes false positives that may arise when RANSAC or the Hough transform is used in isolation. When edge contrast within the YOLOv5-defined ROI is insufficient, and all refined candidates receive low composite scores, a circular Hough-transform fallback is activated within the same region. Hough-derived circles are re-evaluated using the same edge-based and anatomical constraints, and the highest-scoring candidate is retained only if it improves upon the RANSAC-based estimate.

**Figure 5:**
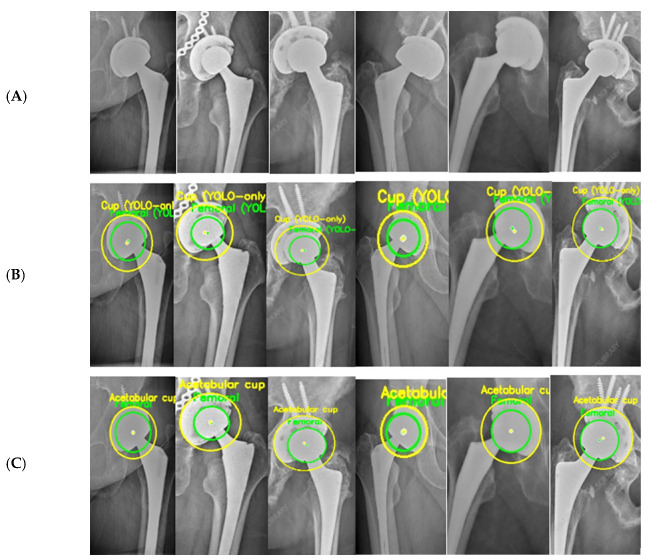
Femoral and AC Circle Detection Outputs on Hip Prosthesis X-ray Images. (**A**) shows the original X-ray images, highlighting the variability in implant designs, image quality, and anatomical configurations. (**B**) presents the intermediate detection results obtained using the YOLOv5-based ROI localization, where coarse circular estimations of the femoral head and acetabular cup are generated. These initial predictions successfully isolate the relevant regions despite challenging conditions such as metallic screws, projection differences, pelvic asymmetry, and partial occlusion of the acetabular rim. (**C**) displays the final refined circle detections after applying the geometric processing pipeline, including edge detection, Edge-Snap refinement, and RANSAC-based circle fitting. The resulting femoral-head and acetabular cup circles exhibit stable center positions and consistent radii across all examples.

Figure 5 provides a qualitative illustration of the proposed framework on real hip prosthesis radiographs from the external test dataset. 

The qualitative results demonstrate the robustness and reliability of the proposed hybrid approach in accurately identifying circular prosthetic components across heterogeneous radiographic conditions. All radiographs shown were exclusively used for testing and were not included in the training or validation stages, confirming the generalization capability of the proposed framework.

### 2.6. Computation of Medial, Superior, and Resultant Wear

Wear was computed by comparing the femoral head and AC circle parameters $\left(c_{x},\;c_{y},\;r\right)$ obtained from the postoperative (PRE) and follow-up (POST) radiographs.

To reliably quantify wear, each patient must have at least two AP hip radiographs: a PRE and a POST, typically obtained months or years after surgery. Medial, superior, and resultant wear components are computed by comparing the femoral head and AC circle positions across these two time points.

#### 2.6.1. Medial Wear (Center-to-Center Distance)

The 2D distance between the centers of the femoral head and the AC was calculated for both time points:

(16) $$d_{PRE}=\;\sqrt{\left[\begin{array}{c} \;{\left(c_{x},AC,PRE\;-\;c_{x},fem,PRE\right)}^{2}+\\ \;{\left(c_{y},AC,PRE\;-\;c_{y},fem,PRE\right)}^{2} \end{array}\right]}$$

(17) $$\;\;d_{POST}=\sqrt{\left[\begin{array}{c} \;{\left(c_{x},AC,POST-c_{x},fem,POST\right)}^{2}+\\ {\left(c_{y},AC,POST-c_{y},fem,POST\right)}^{2} \end{array}\right]}$$

The two radiographs are often acquired under different geometric conditions. Factors such as the patient being imaged at another hospital, changes in radiographic technique, variations in source-to-image distance, detector zoom, and patient positioning can all alter the magnification of the image. As a result, absolute pixel distances measured on the PRE and POST radiographs are not directly comparable.

To eliminate this problem, all measurements are standardized using the femoral head radius as a fixed anatomical reference. The true physical radius of the prosthetic femoral head is constant throughout the patient’s lifetime and cannot change between examinations. Therefore, the ratio of the femoral head radii in the PRE and POST radiographs provides a reliable scale factor (sf) that compensates for differences in magnification and pixel spacing. By applying this scale factor to the POST measurements, both radiographs are expressed in a common geometric scale, enabling accurate comparison of medial and superior migration.

(18) $$sf=\frac{r_{fem},PRE}{r_{fem},POST}$$

Accordingly, medial wear was computed as follows:

(19) $$\;\;\Delta d\;=\;\left|d_{post\;}\times \;sf\;-\;d_{pre}\right|$$

#### 2.6.2. Superior Wear (Vertical Distance Between Superior Points)

The superior point of each circle was obtained by subtracting the radius from the y-coordinate of the center:

(20) $$y_{top},fem=\;c_{y},fem\;-\;r_{fem}$$

(21) $$y_{top},AC=c_{y},AC-r_{AC}$$

The superior joint-space distance for each time point was as follows:

(22) $$g_{pre}=\;\left|\;y_{top},AC,PRE\;-\;y_{top},fem,PRE\right|$$

(23) $$g_{post}=\left|\;y_{top},AC,POST-y_{top},fem,POST\right|$$

The same scaling factor based on the femoral head radius was applied as follows:

(24) $$\Delta g\;=\;\left|g_{POST\;}\times \;\;sf\;-\;g_{pre}\right|$$

#### 2.6.3. Resultant Wear Magnitude

The overall wear magnitude (wear resultant) was computed by combining the medial and superior wear components:

(25) $$\Delta_{total\;wear}=\;\sqrt{\left[\;{\left(\Delta d\right)}^{2}+\;{\left(\Delta g\right)}^{2}\right]}$$

All computed values (femoral head radii, scale factor, center distances, superior gaps, Δd, Δg, and $\Delta_{total\;wear}$) were saved in text format, producing a standardized quantitative report for each patient and enabling further statistical analysis. The resulting wear values are computed in pixels, and if the radiographic magnification factor is known, these pixel measurements can be directly converted into millimeters to provide clinically interpretable wear measurements.

## 3. Results

The experimental evaluation was structured to reflect the sequential stages of the proposed pipeline. Since accurate wear measurement depends on robust detection, reliable circle estimation, and consistent longitudinal comparison, the results section is organized into three core components. First, we present the performance of the YOLOv5-based ROI localization module, which identifies the hip prosthesis region and enables all downstream processing. Second, we evaluate the accuracy of the femoral head and AC circle detection in terms of center and radius estimation errors, as these geometric parameters form the basis of the wear computation. Finally, we report the medial, superior, and resultant wear measurements obtained from longitudinal PRE–POST radiographs, including example patient cases and numerical wear outputs. This structure ensures that each stage of the pipeline is validated before interpreting the final wear results.

### 3.1. Detector Performance (YOLOv5 ROI Localization)

The first stage of the proposed pipeline is the automatic localization of the femoral head and AC regions using a YOLOv5-based detector. The detector was trained on the annotated dataset developed for this study. Its performance was evaluated through training/validation loss behavior, mAP metrics, and precision–recall characteristics.

#### 3.1.1. Training and Loss Behavior

**Figure 6:**
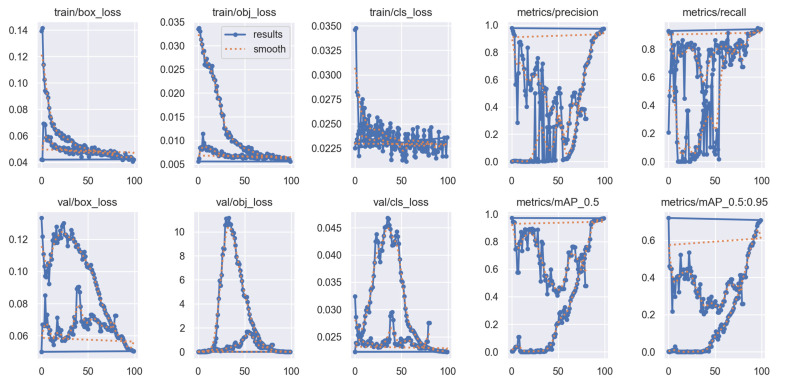
Training and validation loss curves.

As illustrated in Figure 6, the box loss, objectness loss, and classification loss all exhibited smooth and consistent convergence during training. The box regression loss decreased steadily, with the training curve falling from approximately 0.14 to 0.04 and the validation curve decreasing from 0.12 to 0.06, indicating progressively improved alignment between predicted and ground-truth bounding boxes. The objectness loss followed a similarly favorable trend, with the training loss reducing from about 0.03 to 0.005 and the validation loss converging to low and stable values, reflecting increasing confidence in identifying prosthesis-related regions.

The classification loss also improved consistently, decreasing from 0.035 to 0.022 on the training set and from 0.045 to 0.025 on the validation set, which demonstrates enhanced ability to differentiate between the femoral head and AC classes. Taken together, the evolution of all loss components shows strong and stable convergence. The close agreement between the training and validation curves demonstrates that the model maintained excellent generalization performance and did not exhibit signs of overfitting during training. After approximately the 50th epoch, all losses stabilized at low and consistent levels, confirming that the detector learned a reliable and well-balanced representation of the target structures.

#### 3.1.2. Precision–Recall Performance

**Figure 7:**
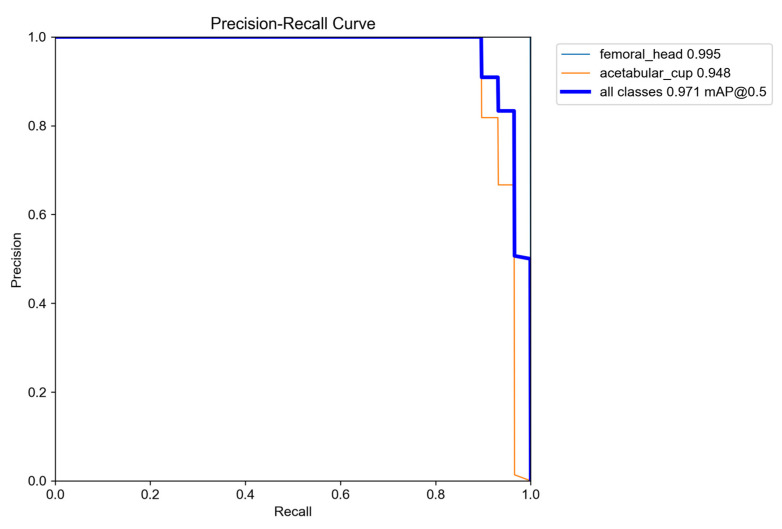
Precision–Recall curves for both classes.

The precision–recall characteristics of the detector are illustrated in Figure 7, which shows class-specific curves for the femoral head and AC, along with the combined performance curve. The femoral head achieved a precision of 0.995 and a recall of 0.98, reflecting highly reliable detection with near-perfect coverage. The AC component reached a precision of 0.948 and a recall of 0.95, demonstrating strong performance despite the greater structural complexity caused by overlapping bone boundaries and metallic screw artifacts. Across the full range of recall values, the curves indicate that the model maintains high precision, particularly for the femoral head, while achieving balanced precision–recall behavior for the AC. The overall detector performance, aligned with the PR curve labeled “all classes 0.971 mAP@0.5”, confirms that the model provides robust and clinically reliable localization suitable for subsequent circle estimation and wear computation. It is important to emphasize that these mAP values reflect only the raw YOLO predictions. The hybrid refinements applied in later stages, including edge-snap, RANSAC, and Hough transform, were not incorporated in this evaluation. Therefore, the reported values demonstrate that the detector achieves strong accuracy even before any geometric enhancement steps are applied.

#### 3.1.3. Optimal Confidence Threshold Analysis (F1–Confidence Curve)

**Figure 8:**
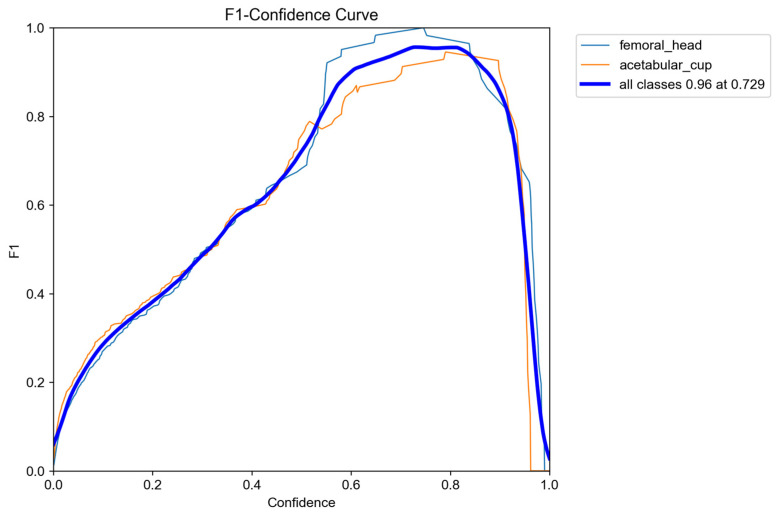
F1-Confidence Curve.

The F1–confidence characteristics of the detector are shown in Figure 8, illustrating how performance varies across different confidence thresholds for both the femoral head and the AC. The curves reveal a broad plateau of high performance, with a peak F1 score of 0.96, demonstrating that the detector maintains excellent localization accuracy across a wide confidence range. The optimal operating point occurred at a confidence threshold of approximately 0.73, which is high for YOLO-based medical image analysis models and reflects the behavior of the raw ROI localization stage before any circle-refinement steps are applied.

In typical radiographic ROI-detection studies, confidence thresholds commonly fall within the 0.60–0.75 range due to the need for suppressing false positives in low-contrast and artifact-prone imaging conditions. Within this context, a threshold of 0.73 reflects a highly selective and well-calibrated detector, capable of identifying true anatomical structures while effectively filtering out noise, overlapping bone shadows, and metallic artifacts. Achieving an F1 score of 0.96 at such a stringent threshold indicates that the model exhibits strong robustness and resilience to common challenges in hip X-ray imaging.

Overall, the consistency of the femoral-head, AC, and overall F1 curves confirms that the detector performs reliably across both anatomical targets, providing a stable and dependable localization stage for subsequent circle fitting and wear computation.

### 3.2. Quantitative Results

Table 2 presents a quantitative comparison between the YOLO-only baseline and the proposed hybrid framework. While the YOLO-only model provides an initial coarse localization, it suffers from high variance and pronounced outliers, particularly in AC center and radius estimation. By integrating geometric refinement, the proposed method substantially improves accuracy across all evaluated metrics. Specifically, the femoral head center error is reduced by approximately 10-fold, while the AC center error shows an improvement of about 4-fold compared to YOLO-only predictions. Similarly, radius estimation accuracy is enhanced by nearly 6-fold for the femoral head and up to 9-fold for the AC. In addition to lowering mean errors, the proposed framework significantly reduces error dispersion and suppresses extreme outliers, resulting in more stable and reliable geometric estimates. The results demonstrate that while YOLO-only provides coarse localization, it suffers from large variance and occasional severe outliers. The proposed hybrid refinement significantly reduces both the mean error and variance for center and radius estimation, confirming the necessity of integrating geometric fitting for precise radiographic measurements.

### 3.3. Circle Detection Accuracy (Femoral Head and AC)

After applying the full refinement pipeline consisting of edge-snap adjustment, RANSAC fitting, and Hough transform, the accuracy of the detected circles was evaluated using normalized metrics. Normalization removes the effects of resolution and magnification differences between radiographs by expressing errors relative to anatomical size, allowing a scale-independent and clinically consistent assessment of center and radius estimation accuracy.

#### 3.3.1. Normalized Center Accuracy

**Figure 9:**
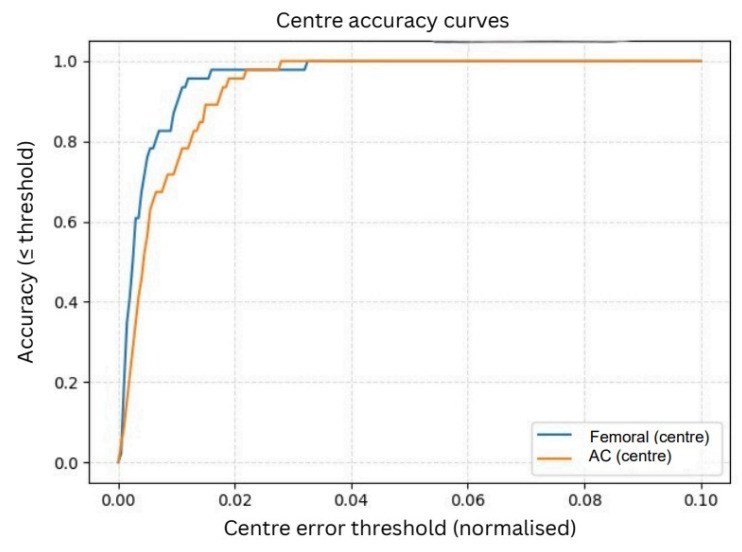
Center accuracy curves.

As shown in Figure 9, the femoral head center demonstrated excellent performance, achieving 95% accuracy at a 1% error tolerance and reaching 100% accuracy at a 2% tolerance. The AC center, although slightly more challenging due to partial rim visibility and overlapping pelvic structures, achieved approximately 90% accuracy at 1% tolerance and increased to around 98% accuracy at 2%. These results confirm that the refined circle centers align very closely with the ground-truth annotations, even under strict error thresholds.

#### 3.3.2. Normalized Radius Accuracy

**Figure 10:**
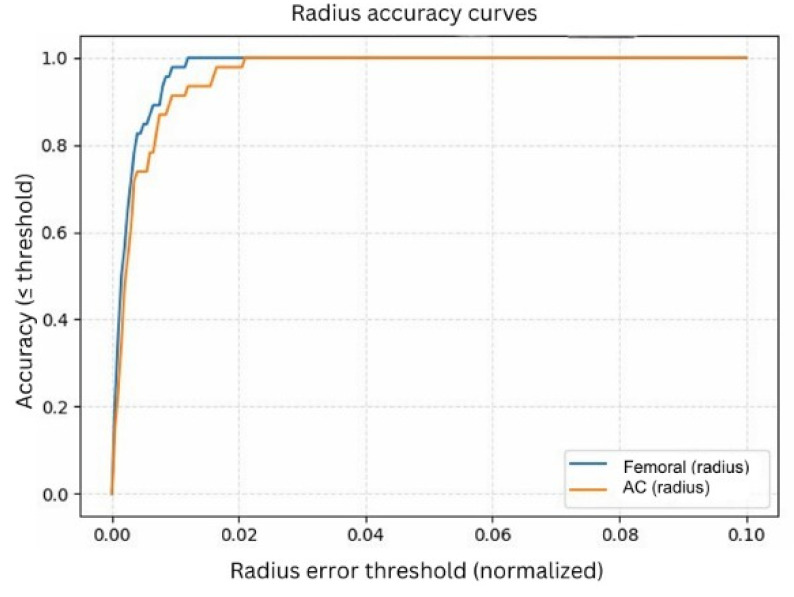
Radius accuracy curves.

The normalized radius accuracy curves (Figure 10) further demonstrate the reliability of the refined circle parameters. Similar to the center analysis, radius errors were normalized to eliminate scale and resolution differences.

The femoral head radius achieved approximately 95% accuracy at a 1% tolerance, while the AC radius reached around 90% at the same threshold. At a 2% tolerance level, both structures converged toward nearly 100% accuracy, indicating that the model consistently estimates anatomically meaningful radii across the dataset. The sharper convergence observed in the normalized curves reflects the advantage of expressing errors relative to anatomical scale rather than absolute pixel distance. Since pixel measurements vary across radiographs due to differences in resolution, zoom, and magnification, pixel-level errors can be misleading. Normalized accuracy provides a more consistent and clinically meaningful evaluation, showing that the model reliably captures the circular geometry of both the femoral head and the AC regardless of image size or acquisition conditions.

### 3.4. Wear Computation Results (Medial, Superior, Resultant Wear)

In this section, the positional changes of the femoral head within the AC were quantified in terms of superior and medial wear. Across the evaluated PRE–POST radiograph pairs, the POST images consistently showed a reduction in the superior joint space together with a medial displacement of the femoral head center. When comparing the PRE and POST radiographs, the differences became visually and numerically evident. The PRE images typically exhibited a symmetric and well-centered femoral head within the AC, whereas the Post images revealed narrowing of the superior gap and a measurable medial shift. Both effects were captured by the automated system, confirming the presence of combined superior and medial wear. Two representative patient cases were analyzed in detail, each consisting of a PRE and a POST radiograph. For both examples, the refined circle outputs were used to compute medial, superior, and resultant wear, and the corresponding numerical values were tabulated for clarity. These tables summarize the full set of wear parameters for each case, including the femoral head radii, scale factor, PRE–POST center distances, vertical gaps, and final wear components. By integrating visual overlays of the detected circles with the associated quantitative measurements, the system provides a comprehensive and interpretable assessment of wear progression.

**Figure 11:**
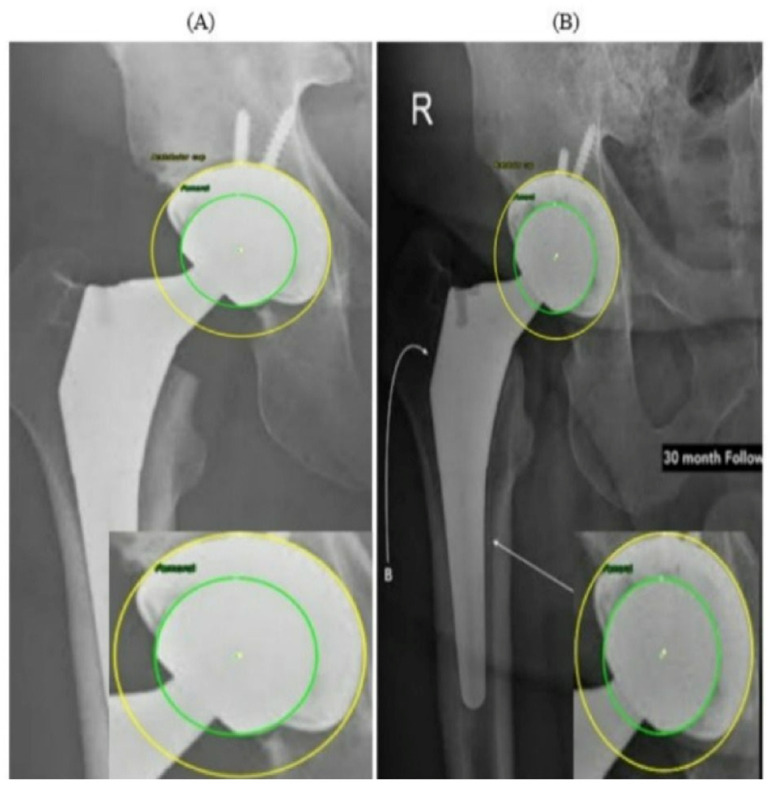
Example 1 of PRE–POST radiographs with detected femoral head and AC circles. (**A**) PRE radiograph demonstrating a centered femoral head within the acetabular cup. (**B**) POST radiograph showing superior displacement of the femoral head consistent with wear progression.

Figure 11 presents two radiographs from the same patient acquired at different time points. In the PRE image (Figure 11A), the prosthetic components appear well aligned, with symmetric and uniform joint spaces surrounding the femoral head. No displacement or asymmetry is observed, and the head is centrally positioned within the AC. In contrast, the 30-month POST radiograph (Figure 11B) shows a subtle superior migration of the femoral head. This displacement produces a clear difference in the superior joint space, together with a relative widening of the inferior gap, indicating superior polyethylene wear. A mild medial shift of the femoral head center is also visible, reflected by narrowing of the joint space on the medial side and confirming the occurrence of medial wear. Thus, the joint space that was initially symmetrical has become asymmetrical over time due to combined superior and medial displacement. This pair of radiographs clearly illustrates the progression of wear over the 30-month period, demonstrating a consistent superomedial direction of femoral head penetration within the AC. Both radiographs in Figure 11 originate from the external evaluation dataset and were not included in the training process.

**Figure 12:**
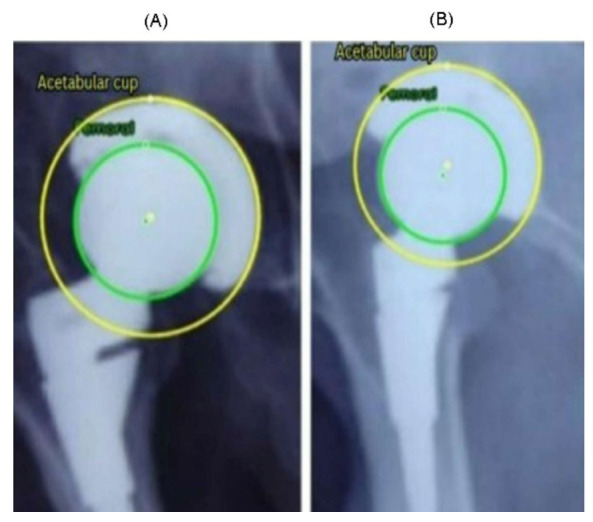
Example 2 of PRE–POST radiographs with detected femoral head and AC circles. (**A**) PRE radiograph demonstrating a centered femoral head within the acetabular cup. (**B**) POST radiograph showing superior displacement of the femoral head consistent with wear progression.

In Figure 12, the PRE radiograph (A) shows the femoral head well-centered within the AC, with a uniform joint space superiorly and medially. The alignment between the two circle centers indicates that no measurable migration has yet occurred. In the POST radiograph obtained several years later (B), the femoral head is positioned closer to the superior aspect of the cup, indicating a clear difference in the superior joint space. This pattern is consistent with progressive superior polyethylene wear. Additionally, a slight medial shift of the femoral head center is visible, demonstrating progressive medial wear over time. Overall, the comparison between (A) and (B) shows a combined superior and medial wear. Similar to Figure 11, the radiographs in Figure 12 were obtained from the external, unseen dataset and were never used for training or validation.

These findings reinforce the value of combining visual and quantitative evidence when interpreting longitudinal THA radiographs. While subtle migration patterns can be difficult to identify through visual inspection alone, particularly when the displacements are only a few pixels, the automated circle-based analysis provides a reproducible framework for detecting even minimal deviations from baseline alignment. The PRE–POST differences captured in Figure 11 and Figure 12 demonstrate how small variations in center position and joint-space geometry accumulate over time to form clinically meaningful wear trajectories. Importantly, the refined circle estimates reduce the influence of image noise, projection variability, and partial rim visibility, ensuring that the observed displacements reflect true anatomical change rather than measurement artifacts. By integrating both geometric overlays and numerical wear metrics, the system enables a clear linkage between qualitative radiographic appearance and quantitative displacement values. This dual-layer interpretation helps distinguish early progressive wear from normal postoperative variation and supports more consistent longitudinal monitoring of implant performance.

Two representative wear cases were quantitatively summarized in Table 3 and Table 4, each including a pair of PRE and POST radiographs along with the automatically computed medial, superior, and resultant wear components. In both examples, the system successfully produced consistent numerical outputs after scale normalization. These examples demonstrate the ability of the proposed framework to extract reliable and fully automated wear measurements. When a magnification factor is available, pixel-based wear values can be converted to millimeters using the following:

(26) $$\;Wear_{\left\{mm\right\}}=\frac{\;Wear_{\left\{px\right\}}}{M}$$

where M  is the pixel-to-millimeter magnification factor. The PRE radiograph is taken as the reference; if the POST image has a different magnification, it is first rescaled to the PRE scale before computing medial, superior, and resultant wear.

## 4. Discussion

The results of this study demonstrate that the proposed hybrid framework can automatically recover the essential geometric parameters required for two-dimensional radiographic wear computation, namely the centers and radii of the femoral head and AC, with robust performance across heterogeneous AP X-ray images. Unlike many existing AI-based approaches that primarily focus on implant classification, loosening detection, or cup-orientation estimation, the present method directly outputs the geometric primitives used in classical 2D wear formulations. From a computational perspective, this distinction is important, since displacement analysis fundamentally depends on the accurate reconstruction of the head–cup geometry across image sequences rather than on high-level categorical predictions.

A key strength of the proposed system is its ability to preserve geometric fidelity during refinement. The normalized center- and radius-accuracy curves exhibit rapid convergence under strict error thresholds, indicating that the refinement pipeline consistently aligns the estimated circles with the true anatomical boundaries, even in radiographs affected by metallic artifacts, partial rim visibility, or low contrast. This behavior suggests that the combination of YOLO-based region localization and deterministic, edge-driven refinement is well-suited to X-ray imaging scenarios characterized by projection variability and heterogeneous image quality, where purely learning-based approaches may struggle to produce stable geometric estimates.

At this proof-of-concept stage, the framework is intentionally limited to automatic ROI localization and the recovery of coarse geometric primitives rather than downstream interpretation or clinical decision-making. For this objective, extremely large datasets are not strictly required. The circular structures of interest exhibit constrained geometric variability, as the femoral head and acetabular cup are designed to approximate standardized spherical and hemispherical forms across implant designs. When combined with extensive data augmentation, the effective variability encountered during training substantially exceeds that of the original dataset, making the available data sufficient for stable localization and reliable geometric reconstruction within the scope of this study.

Unlike traditional systems that require expert-supervised landmark placement, the proposed framework does not rely on expert annotation accuracy at the final stage. The YOLO detector provides only an initial coarse estimate, while the final circles are reconstructed using Edge-Snap constraints, Canny-based boundary guidance, and RANSAC/Hough fitting, which correct small annotation inaccuracies and enforce anatomical consistency.

Following the initial coarse estimate provided by the YOLOv5 detector, the localized region undergoes a refinement stage in which Canny-based edge extraction is applied, non-anatomical gradients are suppressed using an Edge-Snap adjustment, and a robust RANSAC-based circle-fitting procedure is employed to estimate the femoral-head and AC geometry. In cases where rim visibility is limited, a Hough-transform fallback provides an additional geometric prior. Together, these steps refine the detector’s coarse proposal into accurate and anatomically consistent circle estimates suitable for subsequent displacement and wear computation.

The longitudinal examples further illustrate the capability of the framework to capture consistent geometric-displacement trends across image sequences. In the representative cases, the automatically computed wear values reflected the observed superomedial displacement of the femoral head between PRE and POST radiographs. Normalization using the femoral-head radius enabled scale-invariant comparison between follow-up images acquired under different imaging conditions, thereby improving robustness in typical longitudinal imaging scenarios.

Compared with conventional manual and semi-automated wear-computation methods, the proposed framework significantly reduces user dependence and improves repeatability. Classical approaches such as those of Charnley and Livermore rely on manual landmark placement and are sensitive to inter-observer variability. Computer-assisted systems, including those of Martell and Devane, mitigate some of this variability but still require user interaction and operate within rigid software environments. In contrast, the present method is fully automatic, platform-independent, and produces standardized geometric outputs without requiring any user-defined reference points. These characteristics make the framework particularly suitable for large-scale image-analysis pipelines and retrospective or multi-center studies.

Finally, Figure 5 and Table 2 together provide an implicit ablation analysis of the proposed hybrid framework. Figure 5 qualitatively illustrates the progressive refinement behavior from coarse YOLO-based localization to final geometrically constrained circle estimates, while Table 2 quantitatively demonstrates the substantial reduction in error magnitude and variability achieved through geometric refinement when compared with the YOLO-only baseline. The comparative characteristics of manual methods, computer-assisted approaches, and the proposed fully automatic framework are summarized in Table 5, highlighting differences in automation level, user dependence, geometric representation, and computational robustness.

## 5. Conclusions

This study demonstrates that a hybrid deep learning and computer vision framework can robustly and automatically extract circular geometric primitives from anteroposterior X-ray images under challenging imaging conditions. By combining YOLO-based region localization with edge-guided refinement and robust circle fitting, the proposed pipeline produces stable and reproducible estimates of the femoral head and acetabular cup geometry, enabling fully automatic computation of displacement-based wear measures without manual interaction.

The experimental results indicate that the proposed approach can capture consistent geometric displacement trends across sequential radiographs, highlighting its suitability for standardized and observer-independent analysis of circular structures in longitudinal X-ray imaging scenarios. While hip prosthesis wear is employed as a representative case study in this work, the proposed framework is general and applicable to a wide range of X-ray imaging tasks involving circular component detection and geometric measurement.

Future work will focus on extending the proposed model to three-dimensional imaging modalities and integrating radiographic calibration strategies to enable conversion from image-space measurements to physical units when appropriate metadata are available. Such extensions would further enhance the applicability of the framework for large-scale image analysis pipelines and longitudinal studies requiring consistent, automated, and geometry-preserving wear assessment.

## Figures and Tables

**Table 1:** Training strategies with YOLOv5 and their motivation.

Strategy	Aim	Rationale
cosine Learning rate (LR) with warm-up	Accelerate convergence while enabling fine-grained late-stage optimization	Training starts with a relatively high LR to allow rapid adaptation, followed by a gradual cosine decay that yields smaller, more stable updates near convergence. This combination improves both speed and final accuracy compared with a fixed LR.
adaptive moment estimation with weight decay (AdamW)	Balance fast convergence and stability	AdamW combines adaptive per-parameter LRs with decoupled weight decay, allowing progress in flat regions of the loss landscape while maintaining stability in areas with large gradients. This is particularly helpful on small, noisy medical datasets.
Early stopping	Prevent overfitting and save computational resources	Training is terminated when validation performance fails to improve for a predefined patience window, reducing the risk of overfitting to the training set and avoiding unnecessary GPU time once the model has effectively converged.
Label smoothing	Reduce over-confident predictions	Softening the one-hot class targets with a small smoothing factor discourages extreme confidence in individual predictions, improving calibration and robustness to label noise and borderline cases in medical images.
Aspect-ratio–preserving resizing (rectangular training)	Preserve anatomical integrity during rescaling	Images are packed into a 640 × 640 input while preserving the original aspect ratio, ensuring that bony contours and prosthesis geometry are not distorted. This yields more anatomically realistic training samples and stabilizes gradient flow.
Two-stage fine-tuning (freeze/unfreeze)	Retain generic features while adapting to the task	In Stage 1, lower backbone layers are frozen to preserve generic visual features, and only higher detection layers are fine-tuned on hip prosthesis images. In Stage 2, all layers are unfrozen for global refinement. This reduces overfitting.
Data augmentation	Increase diversity and improve generalization	Mild geometric transforms (small rotations, translations, and scale changes) and controlled brightness jitters expose the network to realistic variability in pose and exposure. Mosaic is used at low probability to vary context, whereas aggressive operations such as heavy perspective warping, mixup, and copy–paste are disabled to avoid anatomically implausible samples.
Composite detection loss (box + objectness + class)	Jointly optimize localization, presence, and class identity	The total loss combines a bounding-box regression term (geometric alignment), an objectness term (probability that a prosthesis-related structure is present), and a classification term (femoral head vs. acetabular cup (AC)). Proper weighting of these components yields a balanced training signal that improves both localization precision and correct class assignment.
Resource-aware training (AutoBatch + RAM caching)	Maximize GPU utilization and minimize I/O bottlenecks	The batch size is selected automatically to fit the available GPU memory, ensuring efficient utilization without out-of-memory errors. Caching images in RAM reduces disk Input/ Output (I/O), leading to smoother training and more reproducible wall-clock times across runs.

**Table 2:** Quantitative ablation comparison between the YOLO-only baseline and the proposed hybrid framework (normalized by image size).

Metric (Normalized)	Method	Mean ± Std	Median	P95	Max
Center Error (Femoral)	YOLO-only	0.0431 ± 0.1277	0.0101	0.2304	0.7943
	Proposed (YOLO + RANSAC)	0.0043 ± 0.0055	0.0025	0.0115	0.0321
Center Error (AC)	YOLO-only	0.0267 ± 0.0569	0.0152	0.0263	0.3218
	Proposed (YOLO + RANSAC)	0.0068 ± 0.0064	0.0043	0.0186	0.0280
Radius Error (Femoral)	YOLO-only	0.0208 ± 0.0162	0.0210	0.0408	0.0865
	Proposed (YOLO + RANSAC)	0.0033 ± 0.0038	0.0021	0.0111	0.0167
Radius Error (AC)	YOLO-only	0.0445 ± 0.0329	0.0430	0.1055	0.1388
	Proposed (YOLO + RANSAC)	0.0049 ± 0.0060	0.0027	0.0159	0.0276

Note: Mean ± Std denotes the average error and its variability, Median represents the central tendency, P95 indicates the 95th percentile error, and Max corresponds to the worst-case error.

**Table 3:** Automatic measurements and calculated wear values for Figure 11.

Parameter	PRE (A)	POST (B)	POST Scaled (B→A)	Wear (px)
Femoral head radius (px)	126.310866	123.661418	126.310866	–
Scaling factor (sf) (B→A)	–	–	1.021425	–
Center-to-center distance(medial) (px)	4.976189	10.775240	11.006099	6.029910
Superior joint space (px)	73.828397	73.340661	74.911986	1.083589
Total resultant wear (px)	–	–	–	6.126498

**Table 4:** Automatic measurements and calculated wear values for Figure 12.

Parameter	PRE (A)	POST (B)	POST Scaled (B to A)	Wear
Femoral head radius (px)	74.425153	70.160508	74.425153	–
Scaling factor (sf) (B→A)	–	1.060784	1.060784	–
Center-to-center distance(medial) (px)	6.184893	11.878518	12.600541	6.415648
Superior joint space (px)	43.897086	44.981593	47.715753	3.818667
Total resultant wear (px)	–	–	–	7.466107

**Table 5:** Comparative characteristics of wear-measurement approaches.

Feature/Method	Manual Methods (Charnley, Livermore)	Computer-Assisted Methods (Martell, Devane)	Proposed Method (YOLOv5 + Edge Analysis + RANSAC)
Working Principle	Prosthesis boundaries are measured manually using rulers, calipers, or drawn lines.	The user marks reference points on the AC rim and femoral head; the software performs geometric analysis based on these inputs.	A trained YOLOv5 model automatically detects candidate regions; Canny, Hough, and RANSAC steps refine and select the optimal circles.
User Interaction	High (fully dependent on the operator).	Moderate (requires manual point selection).	None (fully automatic; no manual inputs required).
Automation Level	None	Partial	Fully automatic
Geometric Representation	Line and distance measurements; circular structures are not explicitly modeled.	Circle-based analysis; circle parameters are manually entered by the user.	Direct modeling of circular parameters (center, radius, joint space).
User Dependence	Very high; substantial inter-observer variability.	Moderate; variability depends on the accuracy of selected points.	Very low; consistent and repeatable results across users.
Operating System/Platform	Paper, film, and simple measurement tools.	Mostly Windows-based software with limited algorithmic flexibility.	Python-based; runs on multiple platforms.
Time/Workload	High (measurements are time-consuming).	Moderate (manual marking + software analysis).	Low (fast, fully automatic processing).
Accuracy	Observer-dependent; low–moderate.	Moderate–high, but dependent on manual point marking.	High, particularly reliable in low-contrast or screw-augmented implants.
Standardization	Difficult; measurements vary between users.	Partially standardized based on a predefined workflow.	Standardized outputs: visual overlays + numerical (pixel-based) results.
Scope	2D radiographs, manual measurements only.	Martell: 2.5D (single view + geometric approximations); Devane: 3D (AP + lateral radiographs).	2D radiographs with fully automatic detection and circle-based analysis.
Limitations	User error, low repeatability.	Not fully automatic, limited adjustment of parameters.	Relies on 2D radiographs; cannot capture full 3D structural changes.

## Data Availability

The raw data supporting the conclusions of this article will be made available by the authors on request. All radiographs used in this study were collected from open-access sources as referenced in [36,37,38].
